# A Kinetic-Model-Based Approach to Identify Malfunctioning Components in Signal Transduction Pathways from Artificial Clinical Data

**DOI:** 10.1155/2015/415083

**Published:** 2015-11-29

**Authors:** Xianhua Li, Nicholas Ribaudo, Zuyi (Jacky) Huang

**Affiliations:** ^1^Department of Chemical Engineering, Villanova University, Villanova, PA 19085, USA; ^2^The Center for Nonlinear Dynamics & Control (CENDAC), Villanova University, Villanova, PA 19085, USA; ^3^Villanova Center for the Advancement of Sustainability in Engineering (VCASE), Villanova University, Villanova, PA 19085, USA

## Abstract

Detection of malfunctioning reactions or molecules from clinical data is essential for disease treatments. In order to find an alternative to the existing oversimplistic mathematical models, a kinetic model is developed in this work to infer the malfunctioning reactions/molecules by quantifying the similarity between the clinical profile and the output profiles predicted from the model in which certain reactions/molecules malfunction. The new approach was tested in IL-6 and TNF-*α*/NF-*κ*B signaling pathway, for four abnormal conditions including up/downregulation of single reaction rate constants and up/downregulation of single molecules. Since limited quantitative clinical data were available, the IL-6 ODE model was used to generate artificial clinical data for the abnormal steady-state value shown in two key molecules: nuclear STAT3 and SOCS3. Similarly, the TNF-*α*/NF-*κ*B model was used to obtain the data in which abnormal oscillation dynamic was shown in the profile of NF-*κ*B. The results show that the approach developed in this study was able to successfully identify the malfunctioning reactions and molecules from the clinical data. It was also found that this new approach was noise-robust and that it managed to reveal unique solution for the faulty components in a network.

## 1. Introduction

Mathematical modeling approaches have generated considerable research interest in recent years due to the rising need of a thorough understanding of the behavior of systems in science and engineering, including biological networks [1–3]. More specifically, mathematical models can be used to facilitate the inference of malfunctioning reactions that lead to human diseases from clinical data. For example, approaches based on the Boolean model, which can be developed from well-studied biological reactions, have been widely utilized to determine the critical components whose dysfunction is most likely responsible for a significant impact on the output of signaling pathways [4–7]. These approaches also have the potential to directly localize faulty genes. For an example, faulty genes were detected on the basis of the mismatch between the normal gene sequences and the ones predicted by Boolean models in studying oxidative stress response [8]. Apart from model-based approaches, model-free approaches, or data-driven approaches, are commonly used for the detection of pathological states from “-omics” data based on pattern recognition techniques for patients with gastrointestinal stromal tumors [9, 10] and various neurological diseases [11, 12]. Through comprehensive analysis of “-omics” data obtained from sophisticated technologies such as gene array and mass spectrometry, data-driven approaches hold promise as a novel way to identify disease-specific biomarkers. Although these existing approaches have provided feasible avenues for fault detection in signaling pathways involved in human diseases, they either disregard the transient dynamics (e.g., the Boolean-model-based approaches) or have insufficient details of biological systems (e.g., the data-driven approaches). Approaches based on the Boolean model may not provide accurate solutions for fault diagnosis, as the use of mathematical approximations (either 0 or 1) can cause a coincidence of identical calculation results to occur from different combinations of Boolean variables [8]. Significantly different from the Boolean model, a pure logical-based discrete model which considers a molecule either active or inactive and is unable to capture important intermediate states, the ODE-based kinetic models are able to generate continuous and quantitative profiles of molecule concentrations [13]. Kinetic modeling is therefore a more accurate method for investigating malfunctioning pathways in diseases which can be indicated by the levels of some important molecules that vary significantly from patient to patient. There are many research results supporting the superiority of kinetic models. In a study about the EGFR-ERK signaling pathway, the reaction rate of c-Cbl and EGFR binding was reduced by a factor of 100 or 1000 by regulating an ODE model to mimic the effects of the endocytosis impairment. The active ERK concentration at steady state was measured after treatment with the model-selected concentrations of regulators to reflect the influence of selected regulators [14]. Besides, the influence of malfunctioning molecules not only does reflect the abnormal steady-state values, but also may affect the oscillatory behavior [15–17]. It is reported that a biological network can exhibit diverse dynamic behaviors caused by negative feedback loops, such as long-lasting oscillations [18]. The change in the amplitude of an oscillation profile is a rational indicator of the abnormal situations of a negative feedback loop associated with diseases [19]. Mathematical modeling has become an innovative way of investigating the function of feedback loops in a biological network [20, 21]. Compared to a single molecule or reaction, a feedback loop is an easier target for testing therapy schemes. For example, Araujo and collaborators modeled a signaling cascade with a negative feedback loop and found that targeting serially connecting upstream nodes was most effective for inhibiting cancer cells [20].

Overall, the aforementioned work yields preliminary insights into discovering the functions and connections of important nodes in a signaling pathway by mathematical modeling approaches. However, none of the existing methods combines modeling results with clinical data to directly identify the malfunctioning reactions, molecules, or genes in a biological network for diseases. This motivates us to develop a simulation method that can characterize the malfunction of individual reactions or molecules in a biological network so that the exact malfunctioning reactions/molecules can be identified for patients to receive beneficial therapies that consider the interaction of all the components in the disease-related network [23]. In particular, this work presents a kinetic-model-based approach to infer malfunctioning reactions in signaling pathways involved in acute phase response. More specifically, the profiles for signaling molecules from data are compared to the profiles predicted from kinetic models regarding the malfunction of individual reactions. The specific reaction whose malfunction returns a similar profile to the measured molecules is regarded as the malfunctioning reaction that leads to the abnormal profile shown in clinical data. The IL-6 signaling pathway and the TNF-*α*/NF-*κ*B signaling pathway are used as example pathways in this work, since quantitative models have already been developed for them [22, 24, 25]. The IL-6 pathway model presented by Moya et al., 2011 [22], is used to generate profiles of two crucial proteins in IL-6 signaling pathway, that is, nuclear STAT3 dimmer (STAT3N^*∗*^-STAT3N^*∗*^) and SOCS3, in malfunctioning IL-6 signaling where reactions/preexisting molecules are up/downregulated. Similarly, the most comprehensive model developed for TNF-*α*/NF-*κ*B signal transduction, presented in Huang et al., 2008 [25], is used to generate data for NF-*κ*B in the nucleus with abnormal reaction/protein activities. These two models are used here as they have been validated by experimental data for the accuracy of their prediction. Gaussian white noise is added to the generated STAT3, SOCS3, and NF-*κ*B profiles to produce artificial clinical data, which is then used as reference data to identify the malfunctioning reactions/molecules causing the abnormality shown in these data. The influence from the added noise on the efficacy of the developed approach for detecting malfunctioning reactions/molecules is also evaluated in this work. The results prove that the developed approach is able to identify typical malfunctioning reactions/preexisting molecules from clinical data with substantial noise.

## 2. The Method

### 2.1. The Mathematical Model of IL-6 Signaling Pathway

**Figure 1:**
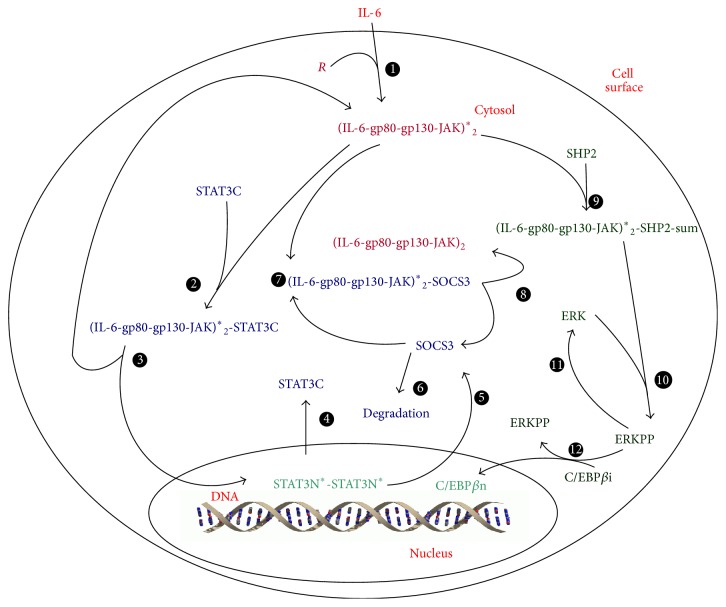
Schematic description of the IL-6 signaling.

IL-6 signaling consists of two pathways, that is, the JAK-STAT and MAPK-C/EBP*β* kinase pathways (Figure 1). It is one of the major pathways that regulate the acute phase response triggered by injuries or infections of the human defensive immune system. The binding of IL-6 to its receptor gp80 on the membrane of hepatocytes activates both JAK-STAT and MAPK-C/EBP*β* pathways in which transcription factors nuclear STAT3 dimer and C/EBP*β* are activated. These two transcription factors in turn regulate the expression of acute phase proteins and other proteins such as SOCS3. SOCS3 is particularly important, as it inhibits the activation of JAKs and thus downregulates both JAK-STAT and MAPK-C/EBP*β* pathways. Hence, SOCS3 initiates a negative feedback loop in IL-6 signaling.

The mathematical model presented in Moya et al., 2011 [22], is the most comprehensive model for IL-6 signaling. It is represented by ODE model (1) with 68 ordinary differential equations (as biochemical reactions), 116 parameters (as reaction constants), and a single input (as the extracellular IL-6 concentration). The model has been validated by experimental data, and it can predict the profiles of 68 intracellular molecules over time. Nuclear STAT3 dimer and SOCS3 are selected as the model outputs, as STAT3N^*∗*^-STAT3N^*∗*^ is a transcription factor and SOCS3 initiates a negative feedback loop. Consider

(1) $$\begin{array}{c} \frac{dx}{dt}=f\left(\;x,p,u\;\right), \end{array}$$

where **x** is a vector of the state variables of the model, **p** is a vector of the parameters, and *u* is the input to the system.

### 2.2. The Mathematical Model of TNF-*α*/NF-*κ*B Signaling Pathway

**Figure 2:**
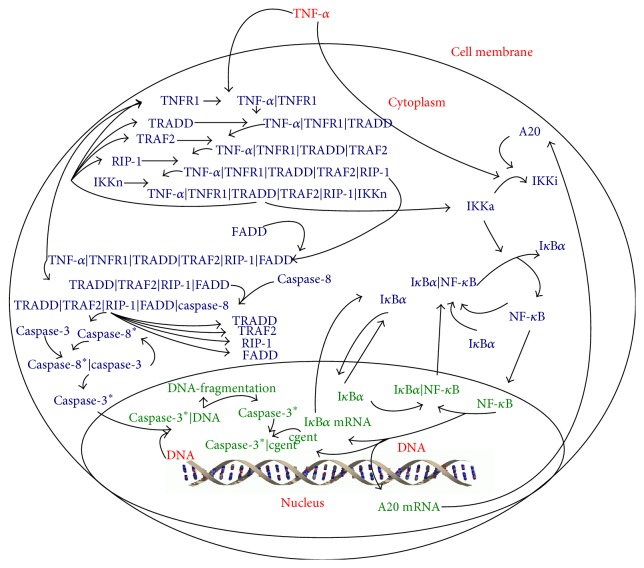
Schematic description of the TNF-*α*/NF-*κ*B signaling.

The TNF-*α*/NF-*κ*B signaling pathway is initiated by the binding of TNF-*α* to its receptor TNFR1 on the cell membrane (Figure 2). The formed binding complex then activates IKKn to IKKa, which releases NF-*κ*B from the complex I*κ*B*α* | NF-*κ*B. The active NF-*κ*B relocates from the cytoplasm into the nucleus and initiates the transcription and translation of I*κ*B*α* and A20, both of which get involved in negative feedback loops that deactivate NF-*κ*B. In particular, I*κ*B*α* binds to active NF-*κ*B in both nucleus and cytoplasm to form complex I*κ*B*α* | NF-*κ*B. A20 deactivates IKKa so that it can not further release NF-*κ*B from the complex I*κ*B*α* | NF-*κ*B. Due to these two negative feedback loops, NF-*κ*B shows sustained oscillation in its time profiles for the liver cells stimulated by TNF-*α*. The model developed by Huang et al., 2008 [25], is used in this work for simulation, as it is one of the most comprehensive models available for the TNF-*α*/NF-*κ*B signaling pathway. Specifically, the ODE model for TNF-*α*/NF-*κ*B signaling consists of 37 ordinary differential equations, 38 parameters, and 16 state variables. The detailed information of the TNF-*α*/NF-*κ*B model can be found in Huang et al., 2008 [25].

### 2.3. The Model-Based Inference Approach to Detect Malfunctioning Reactions/Preexisting Molecules

The irregular activation level of enzymes, either as inhibition or as overexpression, is one of the major reasons causing human diseases. The abnormal concentrations of some important molecules, such as cell signaling receptors on the cell membrane, may also lead to the malfunction of hepatocytes. This work mainly focuses on studying the malfunction due to abnormal activities in single reactions or molecules.

Since clinical data was not available for the hepatocyte cells with the malfunctioning reactions or abnormal protein concentrations studied in this work, artificial clinical data was generated by the following procedure. (1) Extensive simulations for reaction up/downregulation were performed for both IL-6 and TNF-*α*/NF-*κ*B pathways. (2) Abnormal patterns shown in the profiles for nuclear STAT3, SOCS3, and NF-*κ*B were identified. (3) The abnormal pattern that was the most different from the corresponding normal profile was selected as the representative pattern for each of the abnormal conditions that was characterized by the inhibition or overexpression of the enzymes catalyzing single reactions. (4) Gaussian white noise was added to the simulation output for each representative abnormal condition to create artificial clinical data for that condition. In order to generate random noise, the value obtained at each time point from the simulation was used as the mean value of a Gaussian distribution and 20% of the mean value was set as the standard deviation. Monte Carlo sampling approach was used to generate the random noise from the Gaussian distribution. (5) A similar approach was applied to generate clinical data for the abnormal conditions with excessive or deficient preexisting molecules. Therefore, artificial clinical data was generated for four abnormal conditions: two for irregular reaction activities and two for irregular availability of preexisting molecules that have nonzero concentrations prior to the stimulation.

It is our goal to pinpoint the malfunctioning reactions/molecules that cause the abnormality shown in the clinical data. In this work, we developed an approach to infer malfunctioning reactions and preexisting molecules by using a kinetic model to simulate potential malfunctioning conditions (i.e., up/downregulated activity of single reactions and excessive/deficient availability of preexisting molecules). Potential malfunctioning reactions/molecules were found in accordance with the similarity analysis between the model-predicted profiles for the system outputs and those profiles shown in the clinical data. The similarity analysis consists of the following steps.

**Step 1 .**

The profiles for the measured molecules, such as nuclear STAT3, SOCS3, and NF-*κ*B, were produced from the corresponding signaling models in which the rate constant of each reaction was enhanced gradually (e.g., from 5 to 100 with the interval of 5 and then from 100 to 1000 with the interval of 100) to mimic the enzyme overexpression condition. For each reaction rate constant with a certain upregulation level, the model simulated profiles for the measured molecules are compared to clinical data profiles to quantify the similarity defined by the following equation:

(2) S=YclinicalT×YmodelYclinicalYmodel·1Y−clinical/Y−model+Y−model/Y−clinical/2,

where *Y*_model_ is a vector representing the sampled profiles for measured molecules, that is, [**Y**_STAT3_; **Y**_SOCS3_] for IL-6 signaling and **Y**_NF-*κ*B_ for TNF-*α*/NF-*κ*B signaling, generated by the model simulation; *Y*_clinical_ is the counterpart vector from the artificial clinical data; ${\overset{-}{Y}}_{clinical}$ and ${\overset{-}{Y}}_{model}$ are the mean values of the measured molecules over time in the clinical data and model-predicted profiles, respectively; and *S* stands for the similarity between *Y*_model_ and *Y*_clinical_. The first term on the right-hand side of (2) quantifies the shape similarity between *Y*_model_ and *Y*_clinical_, while the second term determines whether *Y*_model_ and *Y*_clinical_ have similar magnitudes. If *Y*_model_ and *Y*_clinical_ are identical in shape, the first term is equal to one. Similarly, if *Y*_model_ and *Y*_clinical_ have the same mean value, that is, ${\overset{-}{Y}}_{clinical}$ equal to ${\overset{-}{Y}}_{model}$, the second term is of a value of one. The value of *S* is within the range of $\left[\begin{array}{cc} 0 & 1 \end{array}\right]$ in which a larger value indicates a higher similarity between the corresponding profiles of *Y*_model_ and *Y*_clinical_. In an ideal situation, only a few *Y*_model_ would show the same abnormal profiles as *Y*_clinical_, resulting in *S* close to the value of one, while any other *Y*_model_ returns an *S* much smaller than one. In this way, the malfunctioning reactions/preexisting molecules can be easily identified because they result in large *S* values.

**Step 2 .**

The approach shown in Step 1 is applied to the other three abnormal clinical conditions, that is, the inhibition of the activity of each individual reaction and the excessive/deficient availability of preexisting molecules. Similar to Step 1, various regulation levels are tested to obtain the maximum possible *S* in these three abnormal clinical conditions. This work mainly focuses on the preexisting molecules with nonzero initial concentrations in the ODE models of the two studied signaling pathways, as these molecules exist before IL-6 or TNF-*α* stimulation and they indicate the pathological state of the patient.

**Step 3 .**

All the inclusive reactions/molecules are sorted by their *S* values quantified for all four abnormal clinical conditions in Steps 1 and 2, in order to identify the malfunctioning reactions/molecules that result in the measured molecules (e.g., STAT3N^*∗*^-STAT3N^*∗*^, SOCS3, and NF-*κ*B) with the profiles that are the most similar to those shown in the artificial clinical data. These malfunctioning reactions/molecules with large similarity *S* values may reveal the real reasons for the artificial clinical data to be abnormal and thus provide directions for the treatment of diseases.

**Figure 3:**
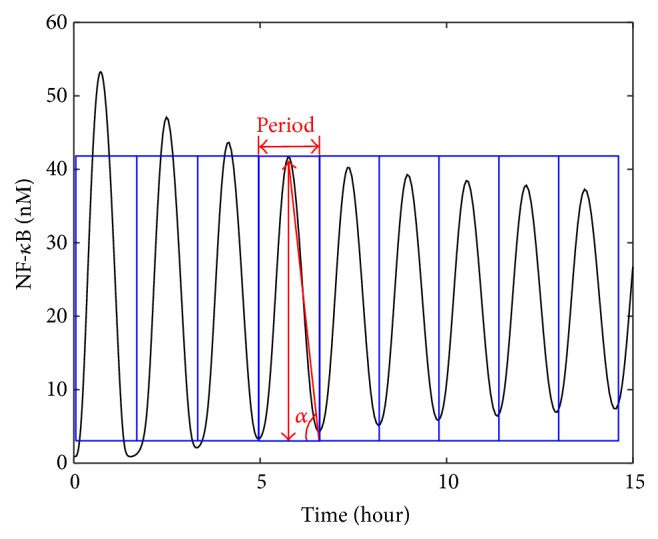
An illustration of factors *R* and *A*. In this example, *N* equals 9, *R* equals tan⁡*α*, and *A* is the total area of the nine identical blue rectangles.

In addition to the measurement of *S* shown in (2) and the aforementioned three steps, two supplemental factors *R* (with the unit of nM/s) and *A* (with the unit of nM·s·10^6^) are defined to achieve better evaluation of oscillation features shown in NF-*κ*B profile in the TNF-*α*/NF-*κ*B signaling model:

(3) R=2HP,

(4) A=HPN,

where *H* is amplitude, *P* is period, and *N* represents number of peak-pairs shown in the oscillation profiles. The steps of calculating *R* and *A* factor are illustrated as follows (Figure 3): (1) all the peak points (maximum and minimum) are determined within the set time (15 hours) along with the total number of pairs of peaks, their corresponding time points, and values; (2) the first minimum peak and the last maximum peak were eliminated from the calculation, as these two points rely on the chosen time range and they may not contain complete peak-pairs; (3) the arithmetic mean values of maximum peaks and minimum peaks are calculated, respectively (as *H*_max_ and *H*_min_), and then the mean amplitude *H* is obtained by (5); (4) the arithmetic mean value of the periods was calculated from two adjacent maximum peaks and two adjacent minimum peaks, respectively (as *P*_max_ and *P*_min_), and the period *P* is determined as the mean of *P*_max_ and *P*_min_ by (6); (5) the value of *A* is calculated by (4) and *R* is calculated by (3). Consider

(5) H=Hmax⁡−Hmin⁡,

(6) P=Pmax⁡+Pmin⁡2.

For better illustration, it is assumed that the calculated results of *H* and *P* coincidentally exist in Figure 3; thus *R* can be also approximated as tan *α*. Therefore, *R* is used to simply reveal the overall shape of an oscillation profile. If two profiles share similar shapes but with an apparent difference in the magnitude, they may be regarded as similar profiles if only *R* factor is taken into account. In such a situation, the *A* factor can indicate the difference shown in the magnitude, as *A* represents the sum of areas within blue rectangles in Figure 3. Two profiles with close *R* values which at the same time have a large difference in the magnitude will probably have a large difference in the *A* factor. In addition to the similarity factor *S* defined in (2), the factors *R* and *A* are taken into account to rank the malfunctioning reactions for the oscillatory output profiles like NF-*κ*B. To determine similar profiles, we mainly consider *S* first for all situations. When oscillation profiles are involved, *R* and *A* can be used as supplemental criteria to further distinguish shape differences. A larger *R* or *A* generally means stronger oscillation.

## 3. The Results

### 3.1. Detection of Malfunctioning Reactions and Molecules in IL-6 Signaling Pathway from Artificial Clinical Data

#### 3.1.1. The Dynamic Patterns of Nuclear STAT3 and SOCS3 in Hepatocyte Cells with Abnormal IL-6 Signaling

**Figure 4:**
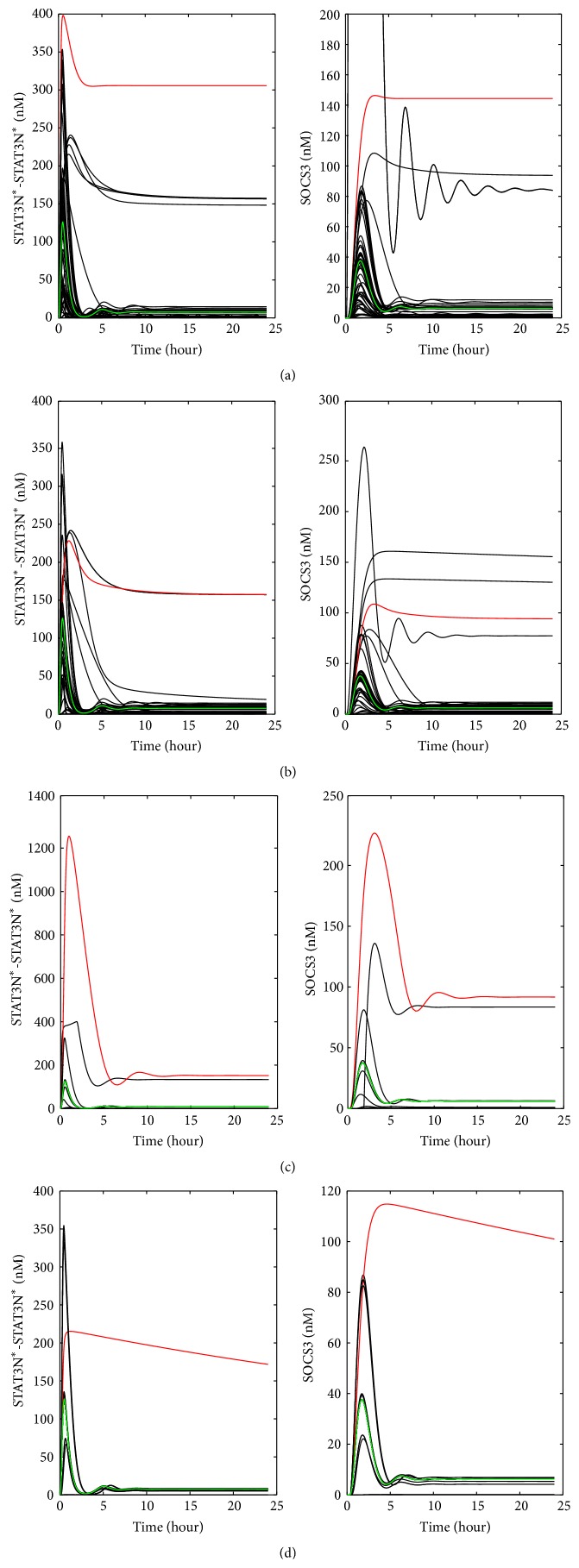
The patterns shown in STAT3N^*∗*^-STAT3N^*∗*^ and SOCS3 problems upon the down/upregulation of single reactions/preexisting proteins: (a) upregulation of each reaction rate constant by 1000 times, (b) downregulation of each reaction rate constant by 0.001 times, (c) upregulation of each preexisting molecule by 100 times, and (d) downregulation of each preexisting molecule by 0.01 times. The red curves represent representative abnormal patterns studied in this work, while the green curves are obtained for the normal condition.

The IL-6 model was used to generate the profiles for STAT3N^*∗*^-STAT3N^*∗*^ and SOCS3 for single reaction upregulation (Figure 4(a)), single reaction inhibition (Figure 4(b)), upregulation of single preexisting molecule (Figure 4(c)), and downregulation of single preexisting molecule (Figure 4(d)). As mentioned above, various up/downregulation levels were applied to each reaction rate constant or preexisting molecule. There are 116 reaction rate constants and 18 preexisting molecules studied in this work. Due to space limitation, Figure 4 only shows the simulation results for one up/downregulation level. For each of the four abnormal clinical conditions, the irregular profiles (marked in red color) that varied most significantly from the normal profiles (marked in green color) were selected as the representative profiles that would be used later to generate the artificial clinical data. The representative irregular profiles were obtained by upregulating reaction Rxn10 (i.e., the red curve in Figure 4(a)) (Rxn10 is the forward reaction of (IL-6-gp80-gp130-JAK)^*∗*^_2_ + STAT3C = (IL-6-gp80-gp130-JAK)^*∗*^_2_-STAT3C), downregulating reaction Rxn44 (i.e., the red curve in Figure 4(b)) (Rxn44 is the forward reaction of (IL-6-gp80-gp130-JAK)^*∗*^_2_ + SOCS3 = (IL-6-gp80-gp130-JAK)^*∗*^_2_-SOCS3), upregulating molecule STAT3C (i.e., the red curve in Figure 4(c)), and downregulating molecule PP2 (i.e., the red curve in Figure 4(d)). These profiles were selected as they had the largest change in the steady-state values for each of the four abnormal clinical conditions.

Upon the upregulation of individual reaction rate constants, Figure 4(a) shows that both STAT3N^*∗*^-STAT3N^*∗*^ and SOCS3 profiles can have significant changes in shape, peak value, and steady-state value. On the other hand, only a few profiles deviate significantly from the normal profiles. They construct a small pool of candidates for identifying the malfunctioning reactions from the clinical data. They are closely related to the activation of STAT3 in the JAK-STAT pathway. An interesting observation from Figure 4(b) is that, as a contradictory abnormal situation to the one shown in Figure 4(a), the inhibition of a single reaction caused similar change pattern in maximum and steady-state level as shown in Figure 4(a). There is a noticeable difference though. For the same scale of change, the magnitude change for the upregulation condition is generally larger than the one for the inhibition condition.

**Figure 5:**
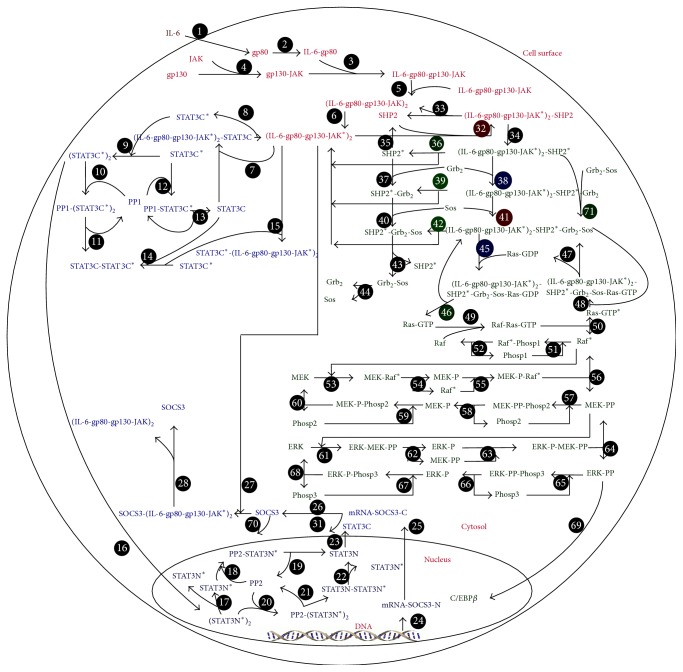
The detail of the IL-6 signaling pathway. It was adapted from Moya et al., 2011 [22].

As shown in Figure 4(c), the upregulation of two preexisting proteins, that is, cytoplasmic STAT (STAT3C) and JAK, leads to a larger increase in the peak values of STAT3N^*∗*^-STAT3N^*∗*^ and SOCS3 than the up- or downregulation of individual reactions shown in Figures 4(a) and 4(b). As a result of downregulating preexisting molecules, most of the shapes shown in STAT3N^*∗*^-STAT3N^*∗*^ and SOCS3 profiles are different from the profiles of the normal condition. The largest change in STAT3N^*∗*^-STAT3N^*∗*^ and SOCS3 profiles shown in Figure 4(d) is due to the inhibition of the preexisting molecule PP2, which is involved in the pathway deactivating nuclear STAT3 dimer (as shown in Figure 5) and bringing it back to the cytoplasm. Downregulating the concentration of PP2 is therefore expected to enhance the nuclear STAT3 dimer, which in turn leads to a higher expression of SOCS3.

**Figure 6:**
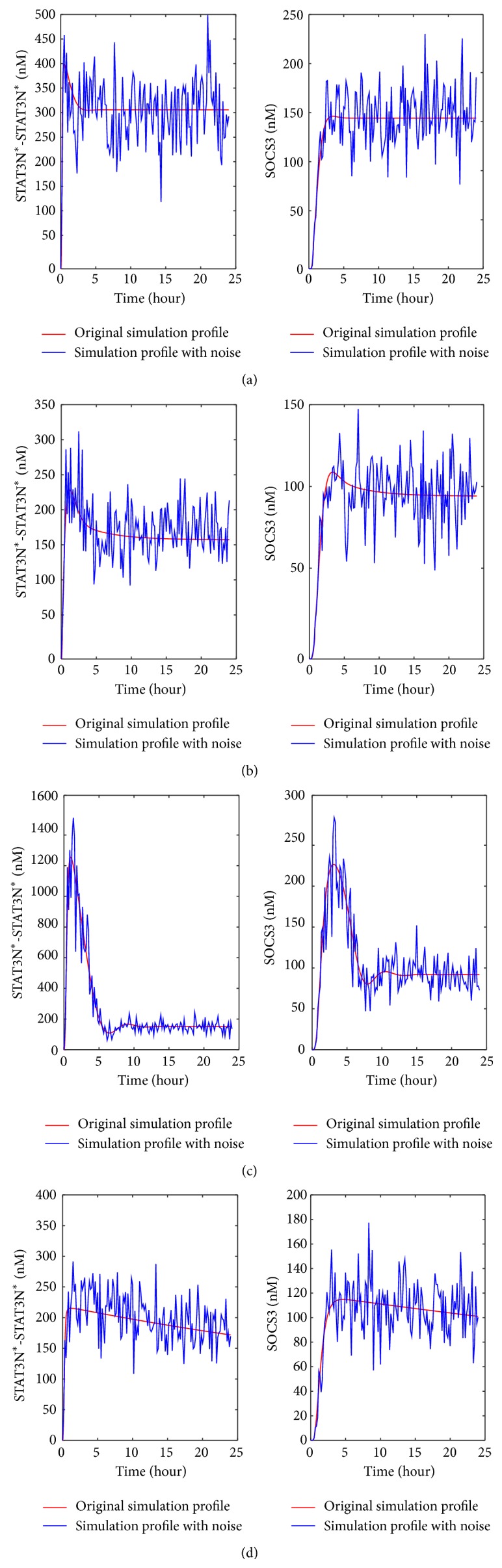
The artificial clinical data generated by adding noise to the nuclear STAT3 dimer and SOCS3 profiles from the model simulation: (a) upregulation of each reaction rate, (b) downregulation of each reaction rate, (c) upregulation of each preexisting molecule, and (d) downregulation of each preexisting molecule.

As mentioned in Figure 5, limited clinical data is available in the literature for abnormal IL-6 signaling. The representative profiles of STAT3N^*∗*^-STAT3N^*∗*^ and SOCS3 obtained in Figure 4 were used to generate the artificial clinical data as described in Section 2.3. Gaussian white noise was added to these representative profiles (the red curves shown in Figure 4) to account for the fluctuation in clinical data. The artificial clinical data (the blue curve) was plotted with the original representative profiles in Figure 6, which shows that the clinical data generally follows the same trends exhibited in the original simulation data.

#### 3.1.2. Detection of Malfunctioning Reactions from the Clinical Data

The developed approach described in Section 2.3 was applied to the clinical data shown in Figure 6 (i.e., the blue curves) to detect the malfunctioning reactions/preexisting molecules that lead to the abnormal nuclear STAT3 dimer and SOCS3 profiles. This section shows the results for detecting malfunctioning reactions from the artificial clinical data, while the next section focuses on the detection of malfunctioning preexisting molecules from the data.

**Figure 7:**
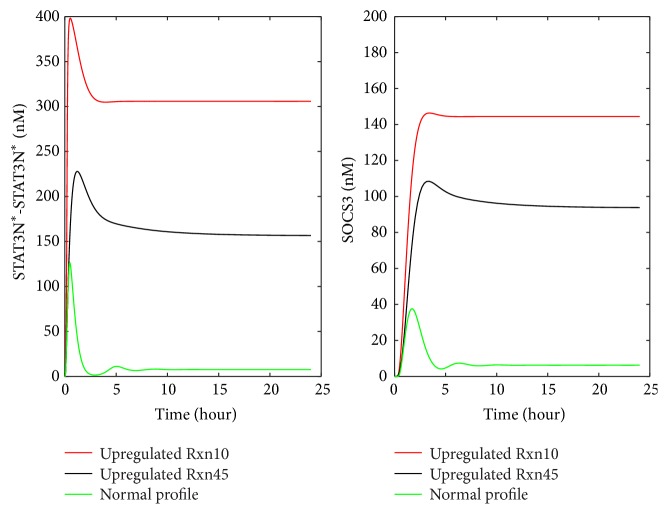
The profiles of STAT3N^*∗*^-STAT3N^*∗*^ and SOCS3 upon the upregulation of Rxn10 and Rxn45.

Table 1 ranks the reactions based on the similarity by combining the nuclear STAT3 dimer and SOCS3 profiles as one-column vectors in *Y*_clinical_ and *Y*_model_ that were obtained by upregulating single reaction rate constants. The ranking that is solely based on the similarity in either the nuclear STAT3 profile or the SOCS3 profile is also listed in Table 1. The combined *S* is calculated by (2) using vector *Y* that contains columns of values for both nuclear STAT3 dimer and SOCS3. The upregulation of either Rxn10 or Rxn45 shows high values of similarity in both the nuclear STAT3 and SOCS3 profiles. On the other hand, the upregulation of other reactions results in small similarity values in both profiles. It is thus straightforward to conclude that the upregulation of Rxn10 or Rxn45 (Rxn45 is the reverse reaction of (IL-6-gp80-gp130-JAK)^*∗*^_2_ + SOCS3 = (IL-6-gp80-gp130-JAK)^*∗*^_2_-SOCS3) accounts for the abnormal profiles shown in the clinical data (i.e., Figure 6(a)). The artificial clinical data for this test was obtained by upregulating the forward rate constant of Rxn10. Since Rxn10 is ranked as the Number 1 malfunctioning reaction in Table 1, it is clear that the developed approach is able to correctly detect the malfunctioning reaction from the data. Rxn10 is the reaction for STAT3C to bind to the receptor complex (IL-6-gp80-gp130-JAK)^*∗*^_2_, which is the first step for the activation of STAT3C to STAT3C^*∗*^. It is expected that it is essential for the activation of STAT3N^*∗*^-STAT3N^*∗*^. On the other hand, Rxn45 is the reaction in which (IL-6-gp80-gp130-JAK)^*∗*^_2_-SOCS3 decomposes into SOCS3 and (IL-6-gp80-gp130-JAK)^*∗*^_2_. SOCS3 inhibits the JAK/STAT pathway by taking (IL-6-gp80-gp130-JAK)^*∗*^_2_ away from binding nuclear STAT3 [24]. If Rxn45 (should this be Rxn10) is upregulated, a greater amount of (IL-6-gp80-gp130-JAK)^*∗*^_2_ is available for activating more nuclear STAT3N^*∗*^-STAT3N^*∗*^. At the same time, more SOCS3 is released from (IL-6-gp80-gp130-JAK)^*∗*^_2_-SOCS3. This explains the increase in both nuclear STAT3 dimer and SOCS3 upon the upregulation of Rxn45. Although the output profiles for upregulating Rxn10 and Rxn45 are similar (as shown in Figure 7), the developed approach can still separate these two reactions, as shown by the difference in the similarity values (i.e., 0.979 for Rxn10 versus 0.832 for Rxn45).

The clinical data for downregulating single reactions (Figure 6(b)) was used to detect the corresponding malfunctioning reaction. The similarity analysis results are shown in Table 2, where Rxn44 is ranked as the top potential reaction to cause the abnormal profiles in the artificial clinical data. Since the data (i.e., Figure 6(b)) was obtained by adding noise to the simulation results after downregulating Rxn44, Rxn44 is expected to be the most likely malfunctioning reaction in this case. An interesting fact is that Rxn44 is the reverse reaction of Rxn45, in which (IL-6-gp80-gp130-JAK)^*∗*^_2_-SOCS3 is formed from SOCS3 and (IL-6-gp80-gp130-JAK)^*∗*^_2_. Therefore, downregulating Rxn44 enhances the concentrations of nuclear STAT3 and SOCS3 (as shown in Figure 5).

#### 3.1.3. Detection of Abnormal Preexisting Molecule Concentrations from the Data

**Figure 8:**
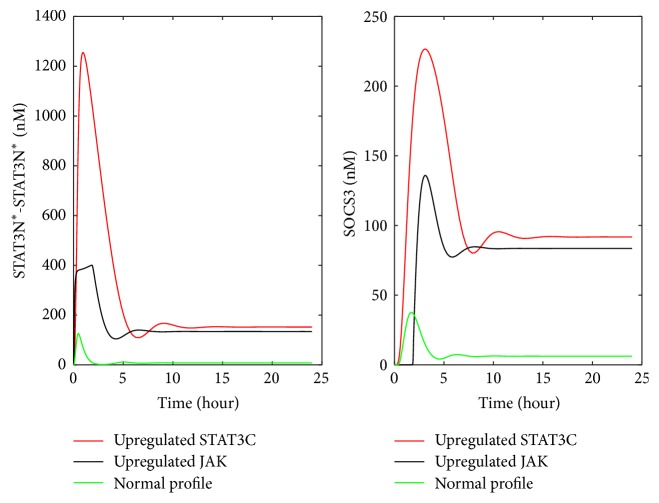
The profiles of nuclear STAT3 dimer and SOCS3 in the hepatocyte upon the upregulation of STATC and JAK.

The artificial clinical data shown in Figure 6(c) was used to detect the corresponding malfunctioning preexisting molecule. Results are shown in Table 3. An enhanced initial condition of STAT3C was ranked as the top driving force for the abnormal profiles of the nuclear STAT3 and SOCS3. Since the artificial clinical data was obtained by enhancing the initial availability of STAT3C, the results in Table 3 prove that the developed approach is able to pinpoint the malfunctioning preexisting molecules from the artificial clinical data. The upregulation of JAK leads to similar changes in the nuclear STAT3 and SOCS3 profiles as those for upregulating STAT3C (Figure 8). In the JAK-STAT3 signaling pathway, JAK is an important component for the activation of the receptor complex (IL-6-gp80-gp130-JAK)^*∗*^_2_, which in turn activates nuclear STAT3 (as shown in Figure 5). This explains the upregulation of JAK concentration results in enhanced concentrations of nuclear STAT3.

The artificial clinical data for downregulating PP2 (Figure 6(d)) were used to identify the malfunctioning preexisting molecule, and the results are shown in Table 4. As expected, PP2 was ranked as the top possible molecule with the irregular initial concentration. The similarity value for PP2 is much higher than any other preexisting molecule. This again proves the accuracy of the developed approach for detecting malfunctioning preexisting molecules.

### 3.2. Detection of Malfunctioning Reactions and Molecules in TNF-*α*/NF-*κ*B Signaling Pathway from Artificial Clinical Data

#### 3.2.1. The Dynamic Patterns of NF-*κ*B from Abnormal TNF-*α*/NF-*κ*B Signaling Pathway

The method of generating artificial clinical data for the TNF-*α*/NF-*κ*B signaling pathway follows the same procedure as the one for IL-6 signaling pathway. Compared to the molecules in IL-6 signaling, the components (e.g., NF-*κ*B) in the TNF-*α*/NF-*κ*B signaling pathway show more complicated and sustained oscillation. Therefore, the factors *R* and *A* defined by (3) and (4) are additionally considered here to further quantify the oscillatory dynamics. Similar to the approach for IL-6 signaling, the TNF-*α* model was used to generate the profiles for NF-*κ*B for four scenarios of abnormal conditions by changing a single component of the network at each run. There are 38 reaction rate constants and 16 preexisting molecules studied in this work. For each of the four abnormal clinical conditions, the irregular profiles (marked in red color) that are significantly different from the normal profiles in oscillation amplitudes and periods were selected as the representative profiles to generate the artificial clinical data. It was identified that the representative irregular profiles were generated by upregulating reaction Rxn9 (i.e., the IKK inactivation reaction caused by A20), downregulating reaction Rxn16 (i.e., the reaction for IKKa | I*κ*B*α* degradation), upregulating molecule cytoplasmic I*κ*B*α* | NF-*κ*B complex, and downregulating molecule TRADD.

**Figure 9:**
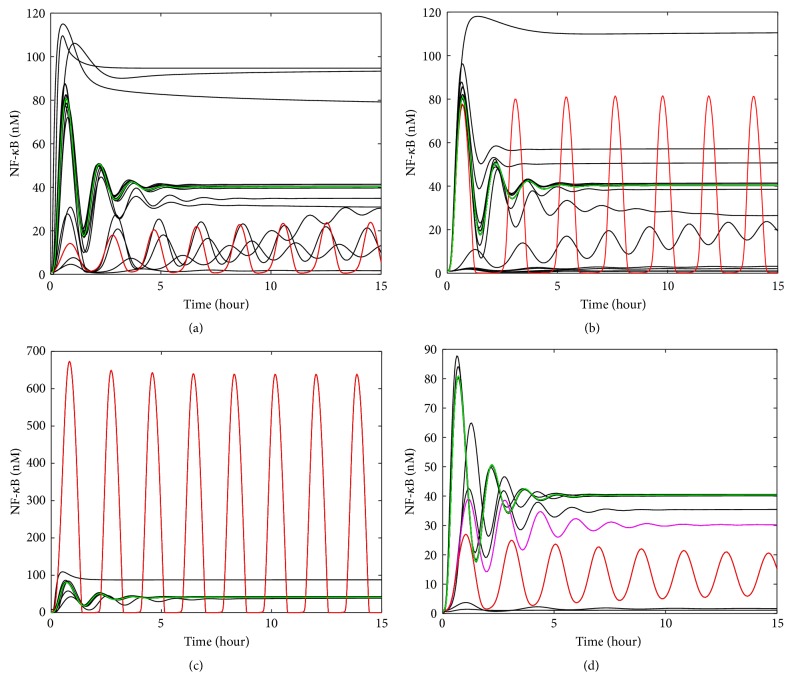
The patterns shown in NF-*κ*B profiles upon the down/upregulation of single reactions/preexisting proteins: (a) upregulation of each reaction rate constant by 100 times, (b) downregulation of each reaction rate constant by 0.0001 times, (c) upregulation of each preexisting molecule by 10 times, and (d) downregulation of each preexisting molecule by 0.01 times. The red curves represent representative abnormal patterns studied in this work, while the green curves are obtained for the normal condition. The magenta curve in (d) is a representative medium level change profile that is used to generate clinical data for Table 10.

Upon the upregulation of individual reaction rate constants, as shown in Figure 9(a), NF-*κ*B profiles have noticeable changes in shape, peak value, and steady-state value. Most importantly, the profile type can shift to be a sustained oscillation curve. The profiles that change most significantly are key influential reactions for the activation of NF-*κ*B in the pathway. The inhibition of single reactions causes similar patterns (Figure 9(b)). However, when compared to the profiles with the same scale of change (i.e., comparing downregulation of ×0.1 times with upregulation of ×10 times, ×0.01 with ×100, and ×0.001 with ×1000), downregulations generally result in the oscillation with higher magnitudes than upregulations (results not shown). These results mean that, in the model of TNF-*α*/NF-*κ*B pathway, downregulations and upregulations of reactions generally influence NF-*κ*B activation with different levels regardless of the change scales. A potential explanation for this phenomenon is that downregulation tends to disturb negative feedback loops that are closely correlated to NF-*κ*B activation in the network, which coordinates several components in response to the change. In this way, downregulations are more sensitive to changes and therefore lead to more serious changes. As a consequence, the NF-*κ*B concentration goes up and down from its initial value intensely and continuously to generate strong oscillation.

**Figure 10:**
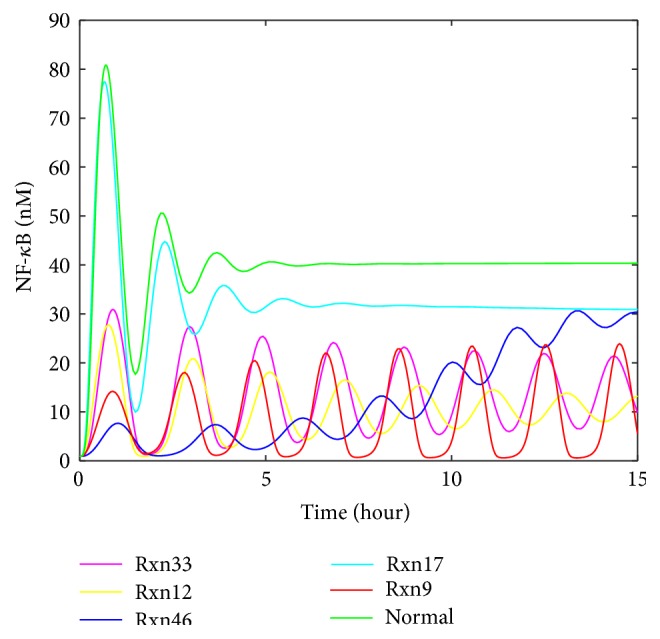
Selected profiles of NF-*κ*B upon the upregulation of single reactions in TNF-*α*/NF-*κ*B signaling pathway.

In Figure 9(a), Rxn9 is IKK inactivation reaction; thus the upregulation of Rxn9 causes the amount of active IKK (i.e., IKKa) to decrease. Therefore, less IKKa acts in the reaction to release NF-*κ*B (Figure 2) from a complex molecule. It is reasonable that the profile with upregulated Rxn9 (Figure 10) tends to have a lower amount of NF-*κ*B. In Figure 9(b), Rxn16 is IKKa | I*κ*B*α* degradation, which is a reaction that has direct impact on the negative feedback loop and production of NF-*κ*B, so the profile with downregulated Rxn16 shows significant oscillation.

As shown in Figure 9(c), the upregulation of cytoplasmic I*κ*B*α* | NF-*κ*B complex can lead to a tremendous change in the magnitude and shape of NF-*κ*B profiles. Cytoplasmic I*κ*B*α* | NF-*κ*B complex is the only source of nuclear NF-*κ*B (as shown in Figure 2), so its amount change certainly will lead to a direct change of NF-*κ*B level. The sustained oscillation profile in Figure 9(d) is due to the inhibition of TRADD. TRADD is involved in the early steps of the activation of TNF-*α* signaling (as shown in Figure 2). Hence, it is expected that downregulating the concentration of TRADD has adverse effect on the TNF-*α* signaling and NF-*κ*B activation.

#### 3.2.2. Detection of Malfunctioning Reactions from the Clinical Data

With the same method as shown in Section 3.1.1, we generated artificial clinical data by adding white noise with the standard deviation *σ* = 0.2.  Table 5 presents the reactions based upon the comparison of factors *S*, *R*, and *A* of NF-*κ*B profiles shown in *Y*_clinical_ and *Y*_model_ that were obtained by upregulating single reaction rate constants. Since *S* factor simply concerns the average difference between pairwise points from the data and model-predicted profiles, *S* may miss some important characteristics of oscillation curve. The factors *R* and *A* were used here to address this issue. Only the most comparable five reactions with the representative reaction data are shown in Table 5 due to the space constraint.

The upregulation of Rxn9 or Rxn33 (i.e., the reaction for the decomposition of TNF-*α* | TNFR1 complex to free TNF-*α* and TNFR1) shows both high values of *S* and similar values of *R* and *A* factors. By contrast, the upregulation of other reactions results in small *S* values and very different values of *R* and *A* factor. It is challenging to distinguish Rxn9 and Rxn33 by visual inspection, as the profiles related to them are very similar. On the other hand, our approach was able to separate these two similar profiles by the *S*, *R*, and *A* factors. Since the artificial clinical data was derived by manipulating the reaction rate constant of Rxn9, it is testified in this case that the developed approach is able to correctly detect malfunctioning reactions from the data. To conclude from this observation highly similar profiles must have high *S* values, whereas high *S* values do not necessarily ensure the similarity in oscillation shapes, because oscillation cases are much more complicated than steady-state cases, in which the pairwise comparison of *S* may leave behind some important features of profiles in a certain period of time. *R* and *A* factor were thus defined as supplemental criteria for describing oscillation features, which suits for this case. Overall, *S* can be used for a rough judgment about similarity in the first place, while adding *R* and *A* to consideration is capable of improving the accuracy of the malfunctioning reaction detection. Therefore, the winning reaction must be selected by higher *S* first and then be identified or confirmed according to reasonable *R* and *A* values that correspond to correct oscillation features.

**Figure 11:**
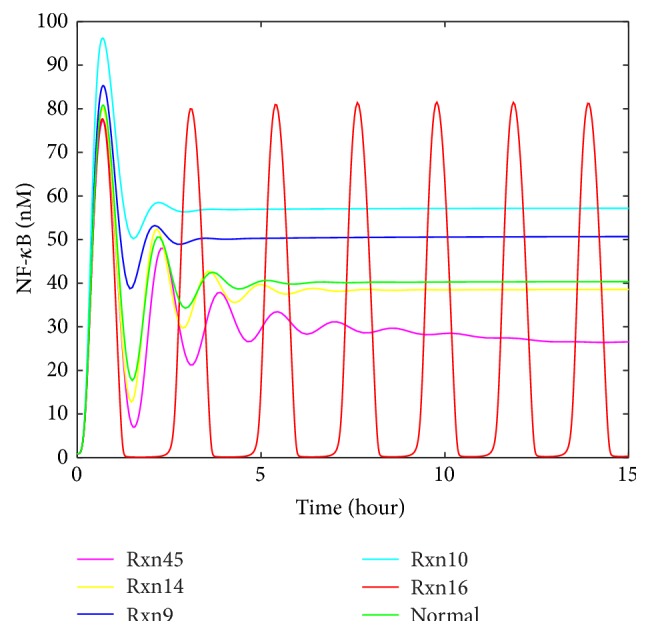
Selected profiles of NF-*κ*B upon the downregulation of single reactions in TNF-*α*/NF-*κ*B signaling pathway.

The analysis result of downregulating single reactions is shown in Table 6. Rxn16 is expected to be the most possible malfunctioning reaction as it is the reaction manipulated to make the artificial clinical data. The result is consistent with the expectation. Both the graph (Figure 11) and the table (Table 6) demonstrated that Rxn16 stood out as the top malfunctioning reaction in this case. Besides, it can be seen that the difference of *S* between the reactions shown in Table 6 is moderate, whereas the differences of *R* and *A* between these reactions can be remarkable. In particular, the *R* and *A* factors directly reflect the shape of every profile and thus make it clearer to distinguish any two profiles. This example supports the superiority of the *R* and *A* factor in the similarity analysis of oscillation profiles.

#### 3.2.3. Detection of Abnormal Preexisting Molecule Concentrations from the Clinical Data

The results of detection of malfunctioning preexisting molecules are shown in Tables 7 and 8. An enhanced initial condition of cytoplasmic I*κ*B*α* | NF-*κ*B is ranked as the top driving force for the abnormal NF-*κ*B profile shown in the artificial data (i.e., the red profile shown in Figure 9(c) with the addition of Gaussian white noise). Since the clinical data was generated by increasing the initial amount of cytoplasmic I*κ*B*α* | NF-*κ*B, the developed approach is able to pinpoint the malfunctioning preexisting molecules from clinical data. While *S* has demonstrated a noticeable difference between cytoplasmic I*κ*B*α* | NF-*κ*B and other molecules, the *R* and *A* factors can provide additional information to indicate more differences between profiles.

The result for the artificial clinical data in which the concentration of TRADD was downregulated is shown in Table 8, which shows that all profiles other than the one for TRADD have small *S* values. This indicates that TRADD can be easily distinguished among all other possibilities for the malfunctioning concentration of preexisting molecules. Even if TRAF2 and RIP-1 have relative big *S* values, their *R* and *A* values are much smaller than those for TRADD. For cytoplasmic A20 and nuclear IkBa | NF-*κ*B, *S* and *A* are very small; however, their *R* values are close to that of TRADD. This case study indicates that the *R* factor may not distinguish two profiles in which the two *α* angles (Figure 3) are of similar value. For this scenario, either *S* or *A* or both can be used to distinguish those profiles.

## 4. Discussion

### 4.1. The Impact of the Added Noise in the Clinical Data on the Detection for Malfunctioning Reactions/Preexisting Molecules

The results shown in Tables 7 and 8 were obtained from the clinical data in which Gaussian white noise was added with a standard deviation (represented by *σ*) equal to 20% of the mean value sampled at each time point from the simulation. When the noise in the data overwhelms the true concentration signal, the profiles shown in the clinical data may not be closely correlative to the model predictions for the four abnormal clinical conditions. In other words, a low similarity value may be obtained for the true malfunctioning reactions/preexisting molecules.

**Figure 12:**
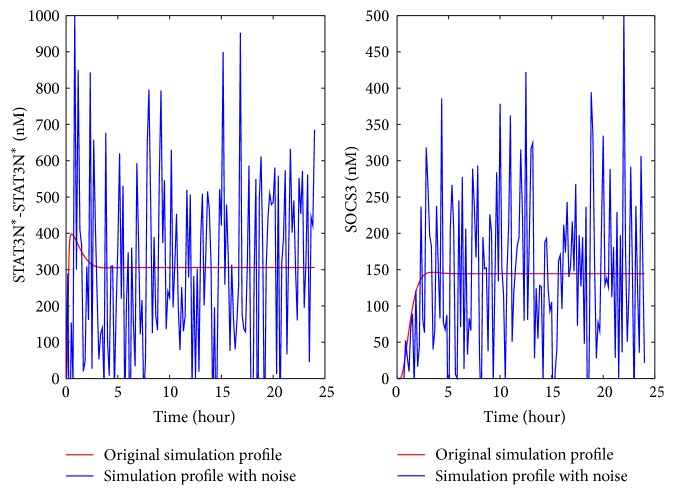
The profiles of nuclear STAT3 and SOCS3 upon the upregulation of Rxn10 with the added noise of the standard deviation *σ* equal to 80% of the mean sampled value.

In order to further evaluate how the noise level in the clinical data influences the accuracy of the developed approach for detecting malfunctioning reactions/preexisting molecules, the developed approach was applied to the clinical data generated for reaction upregulation (e.g., Figure 6(a)) in IL-6 signaling pathway with four different standard deviations, that is, *σ* equal to 20%, 40%, 60%, and 80% of the mean sampled value. Due to the space limitation, only the data for *σ* equal to 80% of the mean sampled values are shown in Figure 12. The comparison between the data in Figures 6(a) and 12 shows that the noise level in Figure 12 is significantly higher.

Table 9 shows the ranking result for the clinical data with different noise levels. It seems that a higher level of noise reduces the similarity values. However, the positions of top-ranked malfunctioning reactions were not changed even though more noise was added to the clinical data. This indicates that the developed similarity analysis approach is noise-robust. Similar results were obtained for the other three abnormal clinical conditions and in TNF-*α*/NF-*κ*B pathway. Due to the space limitation, these results are not shown here.

### 4.2. Potential Multiple Solutions for the Malfunctioning Reactions/Preexisting Molecules

As shown in Table 1 and Figure 7, the upregulation of Rxn10 and Rxn45 shows similar value in the similarity analysis. In addition, Table 2 shows that the downregulation of Rxn44, which is the reverse reaction of Rxn45, leads to similar influence on the nuclear STAT3 dimer and SOCS3 profiles as the upregulation of Rxn45 and Rxn10. If large noise exists in the clinical data, it is possible that these malfunctioning reactions return the same similarity values so that multiple solutions exist. Moreover, the results of different reactions with different regulation levels may return similar results, and the current approach is not able to show the level of regulations. It would be beneficial to distinguish profiles with different regulation levels. In order to achieve this, more variables as the measured outputs should be added, and the scope of the regulation levels should be narrowed down. Due to the complexity of the following work, it is not included in this work. In addition, the malfunction of multiple reactions/preexisting molecules may result in similar change in the profiles of STAT3N^*∗*^-STAT3N^*∗*^ and SOCS3. Due to the space limitation, this work does not study the influence from multiple malfunctioning reactions/preexisting molecules on the cell behavior. The unique solution problem is an interesting topic for further investigation.

One way to address the multiple solution issue is adding more system outputs in the similarity analysis. In this work, only nuclear STAT3 and SOCS3 were monitored and used for the fault detection. Adding other molecules can improve the accuracy of fault detection, as these malfunctioning reactions/molecules that lead to the same change in the profiles of nuclear STAT3 and SOCS3 may result in different change in other signaling molecules. Since monitoring additional signaling molecules is more expensive, it is thus necessary to determine the optimal number of system outputs for measuring. This is another interesting topic for further investigation.

**Figure 13:**
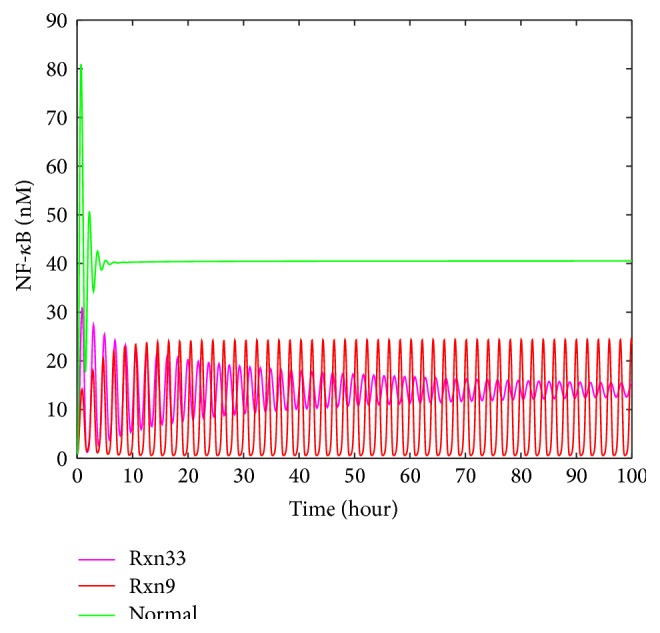
The profiles of NF-*κ*B upon the upregulation of Rxn9 and Rxn33 within 100 hours.

In TNF-*α*/NF-*κ*B pathway, Table 5 and Figure 11 show the similarity of Rxn9 and Rxn33 in the scenario of 100-fold upregulation. Our approach is still able to separate them. As discussed previously, different reactions show different patterns in response to different malfunction levels. Although Rxn9 and Rxn33 seem very similar in the first 15 hours of NF-*κ*B, they gradually show different dynamics in a longer term (Figure 13). Using the profiles for a longer time period is a feasible way to distinguish similar profiles.

### 4.3. The Degree of Difference between the Clinical Data and Normal Profile

In previous discussion, clinical data are corresponding to the most different abnormal profiles. However, sometimes there is a variety of clinical data dealing with not necessarily the most different cases. In order to test our approach on these cases, abnormal cases with medium degree of difference are selected to generate clinical data. Then noise with the standard deviation *σ* equal to 20% of the mean sampled value was added. The same procedure for calculating *S*, *R*, and *A* was utilized.  Table 10 shows an example of investigating the ability of our approach to detect the malfunctioning molecule by downregulating molecules by a factor of 0.01. It is reasonable to see that detecting the malfunctioning reactions for the case with medium degree of difference is challenging. The minor differences among *S* values make it hard to distinguish the possible malfunctioning molecules. However, *R* and *A* turned out to be reliable criteria in this case, and based on them, the correct malfunctioning molecule can still be identified.

### 4.4. The Impact of Data Density on the Detection for Malfunctioning Reactions/Preexisting Molecules

**Figure 14:**
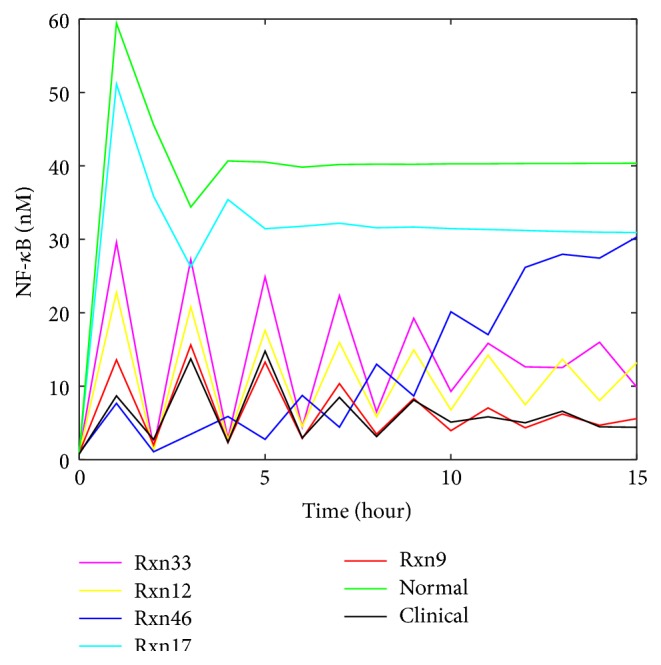
Selected profiles of NF-*κ*B upon the upregulation of single reactions in TNF-*α*/NF-*κ*B signaling pathway. Standard deviation *σ* of noise in clinical data is equal to 20% of the sampled value.

Considering that the clinical data density is possibly rather rare on the time scale compared to the data we used in our approach (six points per hour), the same approach with a lower data density (one point per hour) is investigated here. The results are shown in Table 11 and Figure 14. With this lower data density, although profile features do have some variations, it is still possible for the profiles generated to show clear patterns, and the malfunctioning reactions/molecules can still be identified correctly. This example proved that within a certain range of data density, our approach is still feasible.

### 4.5. Limitation and Future Work

#### 4.5.1. Multiple Malfunctioning Reaction/Molecule Case

The regulation of a single reaction/molecule means the change of the signaling pathway is originated from a single point. This regulation may further lead to change in the downstream reactions in a signal transduction cascade, as signaling reactions highly interact. The developed approach in this paper aims to determine which reactions/molecules are crucial in the signaling pathway of interest to cause abnormal dynamics of the target signal molecule. Only if the effect of single reaction/molecule malfunction is well studied can multiple reaction/molecule malfunction situation be analyzed thoroughly. Existing study shows that a large number of human diseases are associated with the malfunction of a single reaction/molecule. For example, the cognate receptor of TNF, that is, TNFR1, plays a critical role in the control of CNS demyelination and inflammation [26]. Reference [27] lists 13 example human diseases that are associated with the malfunction of a single gene in NF-*κ*B signaling. The emergence of double malfunctioning reactions/molecules in TNF*α*/NF-*κ*B signaling pathway was studied in this work. The results are not shown here due to the space limitation. Preliminary simulation results indicate that the crucial reaction/molecule that generates a significant malfunctioning pattern generally dominates the final malfunctioning dynamics when it is paired with another noncrucial reaction/molecule. Therefore, it can be assumed that it is possible that only a few crucial reactions/molecules have apparent influence in a multiple reaction/molecule malfunction situation. Besides, the combinations of different malfunctioning reactions/molecules may return similar results, thus requiring more variables as measured outputs to further distinguish them. Since a large number of combinations would increase the simulation work heavily, an efficient way is needed to solve this problem. This interesting problem will be further investigated in the future.

#### 4.5.2. Uncertain Noise in Cell Signaling

In any study where clinical data simulation is involved, data noise is the essential part of the study. In this first-trial work, Gaussian white noise was added to the output of the model with nominal parameters and initial values representing a normal person. Only noise from experimental measurement was considered in this work. There may be some difference in the cell behaviors from cell to cell in the human body and among different population of patients due to stochastic properties of signaling pathways and genetic reasons. In order to consider the noise coming from these factors, adding white noise to the model parameters or initial values of considered variables is a potential way to reflect the cell-to-cell and patient-to-patient differences. However, since there are more than 100 parameters and initial variable values in our models, the sampling of outputs and the comparison of output distributions to identity the malfunctioning reactions will be challenging at this time. This is the major reason for only considering measurement noise in this work. To narrow down the uncertainties in the model, the signaling model should be refined from the real data for a specific community of people to narrow down the individual differences.

#### 4.5.3. Stochasticity in the Cell Signaling

The kinetics of individual cells may be affected by stochastic processes in signaling pathways, such as the stochastic gene switching and stochastic receptors activation shown in Lipniacki et al., 2007 [28]. In our approach, we try to use different values of a reaction rate constant to represent the stochastic property shown in a single reaction. We ignore the stochastic properties in the reactions other than the target reaction for malfunction simulation. While including the stochastic properties of other reactions may improve the accuracy of the prediction, the stochastic simulation is computationally intensive for large biological models and it also leads to a distribution of each output value over time (i.e., for multiple cells). Approaches to perform stochastic simulation, distinguish the distributions showing the outputs, and then identify the malfunctioning reactions require further investigation.

## 5. Conclusion

This work demonstrates the first kinetic-model-based approach to detect the malfunctioning reactions/preexisting molecules in a biological network from the artificial clinical data. Four abnormal clinical conditions in two popular signaling pathways involved in acute phase response (i.e., IL-6 and TNF-*α*/NF-*κ*B signaling pathways), including the up/downregulation of single reaction rates and the up/downregulation of single preexisting molecules, were investigated. The simulation results showed that the developed approach was able to successfully detect the malfunctioning reactions/preexisting molecules from the clinical data with a high level of noise. In addition to being robust to noise, the developed approach was able to return a unique solution for fault detection from the clinical data for two signaling molecules (i.e., nuclear STAT3 and SOCS3) in IL-6 signaling pathway and one molecule (i.e., NF-*κ*B) for the TNF-*α*/NF-*κ*B pathway. Although the developed approach was applied to the artificial clinical data, significant noise was added to the data to validate our approach. In addition to these two pathways studied in this work, the developed approach can be applied to other biochemical systems if experimentally validated models are available for them.

## Figures and Tables

**Table 1:** The rank of the possibility of the reactions whose upregulation causes the abnormal profiles shown in Figure 6(a). *S* is the similarity factor determined by (2) from the nuclear STAT3 dimer and SOCS3 profiles.

Rank	Reaction index	*S*	*S* (STAT3N^*∗*^-STAT3N^*∗*^)	*S* (SOCS3)
1	10	0.98	0.98	0.98
2	45	0.83	0.80	0.90
3	46	0.58	0.81	0.0012
4	47	0.57	0.80	0.0013
5	41	0.55	0.78	0.0016
6	35	0.12	0.11	0.18
7	40	0.11	0.0010	0.13
8	43	0.11	0.0010	0.13
9	12	0.10	0.080	0.16
10	9	0.079	0.063	0.13

**Table 2:** The rank of the possibility of the reactions whose downregulation causes the abnormal profiles shown in Figure 6(b).

Rank	Reaction index	*S*	*S* (STAT3N^*∗*^-STAT3N^*∗*^)	*S* (SOCS3)
1	44	0.98	0.98	0.98
2	43	0.77	0.97	0.0017
3	40	0.77	0.97	0.0017
4	47	0.46	0.015	0.88
5	46	0.42	0.014	0.94
6	36	0.29	0.27	0.34
7	41	0.29	0.0040	0.86
8	42	0.25	0.41	0.036
9	34	0.22	0.20	0.27
10	11	0.17	0.15	0.24

**Table 3:** Ranking of the possibility of preexisting molecules whose upregulation causes the abnormal profiles shown in Figure 6(c).

Rank	Reaction index	*S*	*S* (STAT3N^*∗*^-STAT3N^*∗*^)	*S* (SOCS3)
1	STAT3C	0.99	0.99	0.98
2	JAK	0.76	0.74	0.86
3	Grb_2_	0.11	0.091	0.16
4	MEK	0.066	0.053	0.12
5	gp180	0.064	0.051	0.12
6	Phosp2	0.064	0.051	0.12

**Table 4:** The rank of the possibility of preexisting molecules whose downregulation causes the abnormal profiles shown in Figure 6(d).

Rank	Reaction index	*S*	*S* (STAT3N^*∗*^-STAT3N^*∗*^)	*S* (SOCS3)
1	PP2	0.98	0.98	0.98
2	SHP2	0.087	0.078	0.13
3	Gas-GDP	0.086	0.077	0.13
4	Sos	0.086	0.076	0.12
5	Grb_2_	0.085	0.075	0.12
6	PP1	0.081	0.066	0.12

**Table 5:** The top five reactions whose upregulation may cause the abnormal profiles shown in Figure 9(a) (Rxn9 is IKK inactivation reaction caused by A20; Rxn33 is decomposition of TNF-*α*|TNFR1 complex to free TNF-*α* and TNFR1; Rxn12 is IKKa, IKKn, and IKKi degradation; Rxn46 is composition of TNF-*α*|TNFR1|TRADD|TRAF2|RIP-1 and FADD to a complex; Rxn17 is IKK|I*κ*B*α*|NF-*κ*B decomposition with NF-*κ*B releasing).

Number	Reaction index	*S*	*R*	*A*
1	9	0.99	0.0014	0.97
2	33	0.82	0.0015	1.0
3	12	0.69	0.00089	0.68
4	46	0.56	0.00032	0.24
5	17	0.38	0.0010	0.73

**Table 6:** Ranking of the possibility of the reactions whose downregulation causes the abnormal profiles shown in Figure 9(b) (Rxn16 is IKKa|I*κ*B*α* degradation; Rxn45 is release of IKK from IKK|I*κ*B*α*|NF-*κ*B complex; Rxn14 is IKKa and I*κ*B*α* association; Rxn9 is IKK inactivation reaction caused by A20; and Rxn10 is IKK spontaneous inactivation).

Number	Reaction index	*S*	*R*	*A*
1	16	0.98	0.0051	3.8
2	45	0.61	0.0012	0.68
3	14	0.57	0.0012	0.55
4	9	0.50	0.0018	0.25
5	10	0.46	0.0016	0.25

**Table 7:** Rank of the possibility of preexisting molecules whose upregulation causes the abnormal profiles shown in Figure 9(c).

Number	Molecule index	*S*	*R*	*A*
1	Cytoplasmic I*κ*B*α*|NF-*κ*B	0.98	0.048	30
2	TNFR1	0.46	0.00	0.00
3	Free nuclear NF-*κ*B	0.25	0.0011	0.49
4	Free cytoplasmic I*κ*B*α*	0.24	0.0014	0.50
5	Nuclear I*κ*B*α*|NF-*κ*B	0.24	0.0013	0.45

**Table 8:** Rank of the possibility of preexisting molecules whose downregulation causes the abnormal profiles shown in Figure 9(d).

Number	Molecule index	*S*	*R*	*A*
1	TRADD	0.98	0.0014	0.94
2	TRAF2	0.67	0.00062	0.34
3	RIP-1	0.60	0.00048	0.25
4	Cytoplasmic A20	0.50	0.0013	0.47
5	Nuclear I*κ*B*α*|NF-*κ*B	0.50	0.0013	0.46

**Table 9:** Rank of the possibility of the reactions whose upregulation causes the abnormal profiles with different noise levels.

Rank	20%		40%		60%		80%	
	Reaction index	*S*	Reaction index	*S*	Reaction index	*S*	Reaction index	*S*
1	10	0.98	10	0.92	10	0.85	10	0.80
2	45	0.83	45	0.80	45	0.74	45	0.68
3	46	0.58	46	0.56	46	0.51	46	0.48
4	47	0.57	47	0.56	47	0.51	47	0.48
5	41	0.55	41	0.54	41	0.49	41	0.46
6	35	0.12	35	0.12	35	0.11	35	0.10
7	40	0.11	40	0.10	112	0.086	12	0.079
8	43	0.11	43	0.10	43	0.083	9	0.064
9	12	0.10	12	0.093	40	0.083	43	0.062
10	9	0.079	9	0.075	9	0.070	40	0.062

**Table 10:** Rank of the possibility of preexisting molecules whose downregulation causes the abnormal profiles (magenta colored curve shown in Figure 9(d)). TNF-*α*/NF-*κ*B pathway is used for this example.

Number	Molecule index	*S*	*R*	*A*
1	TRAF2	0.98	0.00062	0.34
2	RIP-1	0.97	0.00048	0.25
3	IKKn	0.93	0.00078	0.29
4	Free nuclear NF-*κ*B	0.89	0.00095	0.35
5	Free cytoplasmic NF-*κ*B	0.89	0.0013	0.45

**Table 11:** The top five reactions whose upregulation by a factor of 100 may cause the abnormal profiles. Data density is changed to one point per hour.

Number	Reaction index	*S*	*R*	*A*
1	9	0.98	0.00054	0.34
2	12	0.82	0.00082	0.51
3	33	0.72	0.0011	0.74
4	46	0.46	0.000061	0.040
5	17	0.32	0.00037	0.26
